# Characterization of nit sheath protein functions and transglutaminase-mediated cross-linking in the human head louse, *Pediculus humanus capitis*

**DOI:** 10.1186/s13071-021-04914-z

**Published:** 2021-08-24

**Authors:** Ju Hyeon Kim, Do Eun Lee, SangYoun Park, John M. Clark, Si Hyeock Lee

**Affiliations:** 1 Research Institute of Agriculture and Life Sciences, Seoul National University, Seoul, Republic of Korea; 2 Department of Agricultural Biotechnology, Seoul National University, Seoul, Republic of Korea; 3 School of Systems Biomedical Science, Soongsil University, Seoul, Republic of Korea; 4 Department of Veterinary and Animal Sciences, University of Massachusetts Amherst, Amherst, USA

**Keywords:** Head louse, Nit sheath, LNSP, Transglutaminase, Cross-linking

## Abstract

**Background:**

Head louse females secrete liquid glue during oviposition, which is solidified to form the nit sheath over the egg. Recently, two homologous proteins, named louse nit sheath protein (LNSP) 1 and LNSP 2, were identified as adhesive proteins but the precise mechanism of nit sheath solidification is unknown.

**Methods:**

We determined the temporal transcriptome profiles of the head louse accessory glands plus oviduct, from which putative major structural proteins and those with functional importance were deduced. A series of RNA interference (RNAi) experiments and treatment of an inhibitor were conducted to elucidate the function and action mechanism of each component.

**Results:**

By transcriptome profiling of genes expressed in the louse accessory glands plus uterus, the LNSP1 and LNSP2 along with two hypothetical proteins were confirmed to be the major structural proteins. In addition, several proteins with functional importance, including transglutaminase (TG), defensin 1 and defensin 2, were identified. When *LNSP1* was knocked down via RNA interference, most eggs became nonviable via desiccation, suggesting its role in desiccation resistance. Knockdown of *LNSP2*, however, resulted in oviposition failure, which suggests that LNSP2 may serve as the basic platform to form the nit sheath and may have an additional function of lubrication. Knockdown of *TG* also impaired egg hatching, demonstrating its role in the cross-linking of nit sheath proteins. The role of TG in cross-linking was further confirmed by injecting or hair coating of GGsTop, a TG inhibitor.

**Conclusions:**

Both LNSP1 and LNSP2 are essential for maintaining egg viability besides their function as glue. The TG-mediated cross-linking plays critical roles in water preservation that are essential for ensuring normal embryogenesis. TG-mediated cross-linking mechanism can be employed as a therapeutic target to control human louse eggs, and any topically applied TG inhibitors can be exploited as potential ovicidal agents.

**Graphical abstract:**

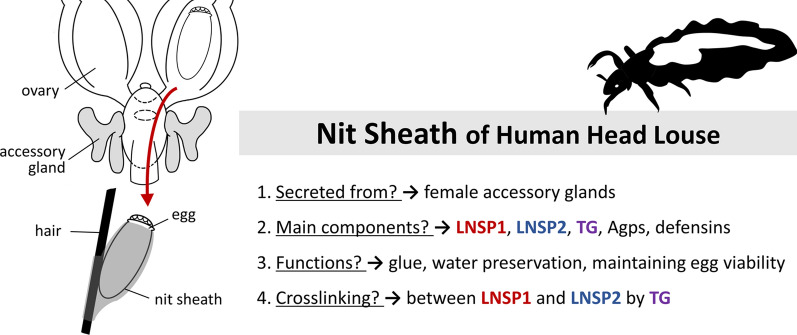

**Supplementary Information:**

The online version contains supplementary material available at 10.1186/s13071-021-04914-z.

## Background

The insect egg is surrounded by an eggshell, a layer formed by secreted proteins and other substances from the follicle cells of oocytes [1]. Eggshells are generally composed of layers of inner vitelline and outer chorionic membranes. The eggshell protects the egg and developing embryo from desiccation, pathogens, parasites and predation. Because sperm, water and air need to pass through specialized domains of the eggshell and embryonic membranes to allow fertilization, metabolism and embryogenesis, insect eggshells have a sophisticated structure [2].

Some insects belonging to the orders Orthoptera, Mantodea and Phthiraptera produce extra materials in order to coat eggs to attach to a substrate and/or to provide additional protection from environment [1]. The materials forming the extra egg sheath (or egg covering) are secreted from the accessory (or collateral) glands of the female’s reproductive organ. Such egg sheath can take various shapes such as a pod (e.g., cockroaches), froth case (e.g., grasshoppers and mantids) or nit sheath (e.g., human lice). The egg sheath proteins exist as liquids within the accessory gland but solidify once secreted during the egg-laying process.

Human head lice (*Pediculus humanus capitis*) cause economic and social problems worldwide whereas human body lice (*P. humanus humanus*) represent a serious public health threat by vectoring several types of bacterial diseases [3]. These lice produce a protective egg sheath, called a nit sheath, to attach newly laid eggs to hair or fabrics. The sheath-forming material, louse glue, is secreted from a pair of female accessory glands connected to the uterus, an expanded part of the common oviduct, during oviposition [4]. In head lice, the gravid females secrete the liquid glue onto the hair shaft first, spread it using the gonopods at the abdominal tip to form suitable oviposition sites, and then place the egg on the oviposition site. Following oviposition, the glue solidifies to form the nit sheath, thereby firmly attaching the eggs to the hair shaft [5]. Histochemical analysis and flash pyrolysis–gas chromatography/mass spectrometry revealed that the nit sheath is composed of proteins and possible aliphatic components cross-linked to each other [6, 7]. Four major protein bands (two with molecular weight of approximately 50 kDa and the other two with molecular weight of approximately 20 kDa) were detected by SDS-PAGE [8], which were similar in size to the composition of body louse nit sheath proteins [5].

More recently, the amino acid composition of the nit sheath proteins was determined, and similarity search against the deduced proteins of the louse genome enabled the identification of two homologous proteins, named louse nit sheath protein (LNSP) 1 and LNSP2, which were suggested to be the previously reported 50 kDa proteins [9]. Along with LNSP1 and LNSP2, N-terminal truncated forms of each protein, LNSP1-like and LNSP2-like, were also identified in the louse genome [9]. Although LNSP1 and LNSP2 appear to be the major structural components of the nit sheath, other minor proteins are also likely involved in the formation of the complex solidified mixture of the nit sheath. Three domains, each characterized by repeating sequences, are found in both LNSP1 and LNSP2. Tandem two-residue repeats of Gln-Ala and Gly-Ala are present in the N-terminal and middle domains, respectively, whereas multiple consecutive Gln residues are found in the C-terminal domain. The N-terminal and middle domains are predicted to fold into β-strands, perhaps further stacking into inter-crossed β filament. LNSP1 and LNSP2 are most predominantly transcribed in the accessory gland of gravid females, demonstrating that these genes actually encode the nit sheath proteins. Although LNSP1 and LNSP2 presumably function as adhesive proteins as determined by recombinant LNSP1 protein, other vital functions, such as resistance to desiccation and defense against pathogenic microorganisms remain to be elucidated [9].

The precise mechanism of nit sheath solidification is unknown. Oxygen exposure appears to be an important triggering factor for the liquid-to-solid curing process [5]. Intermolecular cross-linking between nit sheath proteins, such as LNSP1 and LNSP2, is likely involved as well. Since LNSP1 and LNSP2 contain many Gln residues (86, 18.7% in LNSP1 and 117, 20.9% in LNSP2) and 13 or 12 Lys residues, the covalent cross-linking between ε-amino group of a Lys side chain and γ-carboxamide group of a Gln side chain, which is mediated by transglutaminase (TG), appears to be most feasible among various protein cross-linking mechanisms. Considering that the head louse genome contains one putative TG gene, it is intriguing to speculate that TG may be a promising candidate for regulating the cross-linking of LNSP1 and LNSP2. Additionally, since any uncontrolled cross-linking of nit sheath gel over the operculum of the egg or inside the reproductive system would likely suffocate the egg or hinder the oviposition process, the control mechanisms of cross-linking can be exploited as a novel target for louse control. Knowledge of the cross-linking mechanisms of louse nit sheath proteins may enable the development of compounds that inhibit the solidification process, allowing for their use as novel ovicides and/or to facilitate the physical removal of nits, which are crucial components of treatments for head louse infestations under the “no-nit” policy in schools.

In this study, we determined the temporal transcriptome profiles of the head louse accessory glands plus oviduct, from which putative major structural proteins and those with functional importance, such as proteins necessary for nit sheath cross-linking and microbial defense, were deduced. We then conducted a series of RNA interference (RNAi) experiments to elucidate the function and action mechanism of each component. Knockdown of *LNSP1*, *LNSP2* or *TG* resulted in impairments in oviposition and a dramatic reduction in egg hatch, suggesting all these components were crucial for survival of the embryo. The roles of each component and utilization of TG as a potential therapeutic target for louse infestation are further discussed.

## Methods

### Lice rearing

The South Florida strain of human head lice (SF-HL *Pediculus humanus capitis*) were reared on the in vitro membrane feeding system [10] under environmental conditions of 30 °C and 70% relative humidity and 16/8 h light/dark in a rearing chamber (reviewed and approved by the Institutional Review Board of Seoul National University, IRB No. E1911/003-016).

### Transcriptome analysis

Accessory glands plus uterus and ovaries were dissected from 45 female lice at the emergence day (day 0) or at 5 days after emergence (day 5) in ice-cold, nuclease-free phosphate-buffered saline (PBS; pH 7.4). The tissues were stored separately in RNAlater Stabilization Solution (Invitrogen, Carlsbad, CA, USA) until all tissues were collected. Total RNA was extracted from the tissue with TRI reagent (MRC, Cincinnati, OH, USA) and treated with DNase I (Takara Biotechnology, Shiga, Japan) according to the manufacturer's protocol. mRNA was purified by Oligo (dT) magnetic beads (Qiagen, Germany), and then complementary DNA (cDNA) library construction and adapter ligation was performed with a TruSeq™ Stranded mRNA LT Sample Prep Kit (Illumina, San Diego, CA, USA). Transcriptome sequencing was performed using the NovaSeq 6000 System (Illumina), and quality check on the raw sequences was performed using FastQC version 0.11.7. Reads were trimmed and assembled using the Trimmomatic 0.38 and Trinity programs, respectively. The assembled unigenes were used as queries to search against the National Center for Biotechnology Information (NCBI) non-redundant (NR) protein database using BLASTN of NCBI BLAST version 2.4.0 and BLASTX of DIAMOND version 0.9.21 with an *E*-value default cutoff of 1.0E−5. Reads were mapped to the cDNA sequences using the Kallisto program to calculate the expression level in transcripts per million (TPM).

### Determination of temporal and spatial transcription profiles

In order to quantify the temporal transcription levels of major genes selected from the transcriptome analysis, total RNA was extracted from the dissected accessory glands plus uterus at different ages using TRIzol reagent (MRC) according to the manufacturer’s instructions. In order to confirm the spatial expression sites of major genes, the accessory gland lobes were completely separated from the oviduct and uterus using fine forceps under a stereomicroscope, and total RNA extracted from the separated organs (i.e., accessory gland lobes vs. uterus). Ovaries and alimentary tracts were used as reference organs. First-strand cDNA was synthesized from DNaseI-treated (Takara) total RNA using SuperScript IV Reverse Transcriptase (Invitrogen). Quantitative real-time polymerase chain reaction (qPCR) was performed in the Roche LightCycler 96 system (Roche, Basel, Swiss) using the following cycle conditions: 95 °C for 10 min, 40 cycles of 95 °C for 30 s, 57 °C for 30 s, 72 °C for 30 s, and serial increase of 0.2 °C per 1 s from 45 to 95 °C for melting curve analysis. The reaction mixtures contained 1× TB Green^®^ Premix Ex Taq (Takara) and 0.5 µM primers for target genes (sequences are shown in Additional file 1: Table S1) or ribosomal protein L13A (*RpL13A*) as a reference gene. The relative transcript level of a gene was calculated based on the original concept of 2^−ΔΔCt^ [11]. All qPCR was carried out three times with total RNA extracted independently, and each was conducted with two technical replicates.

### RNAi

Transcription templates of *LNSP1*, *LNSP2*, *TG*, accessory gland protein (*Agp*)*22*, *Agp9* and pacifastin-like serine protease inhibitor (*PSI*) from the cDNA of BR-HL and a pQE30 template from pQE-30 UA vector (Qiagen Korea, Osong, Korea) used as a negative control were generated by PCR amplification using gene-specific primers with the T7 promoter sequence attached at their 5′ ends (Supplementary Table S3). For *LNSP1* and *LNSP2* that show high sequence similarities, respective double-stranded RNA (dsRNA) was designed from gene-specific sites in the N-terminal domains. The PCR products were purified using the Monarch^®^ PCR & DNA Cleanup Kit (New England Biolabs, Ipswich, MA, USA) and then used as templates for dsRNA synthesis with the MEGAscript T7 transcription kit (Ambion, Austin, TX, USA) according to the manufacturer’s instructions. Each dsRNA (138 ng, 69 nl/louse) was injected individually or in combination (in case of *LNSP1* and *LNSP2*) into the second abdominal segments of 2-day-old females using a nano-injector (NanoLiter 2000, World Precision Instruments, FL, USA). pQE30-dsRNA-injected females were used as negative controls. Injected females were returned to hair tufts in the in vitro rearing system with the same number of un-injected males to blood feed. The eggs laid by the females during the period 60 to 84 h after injection were counted and the hatchability of the eggs determined. In parallel, half of the injected females were collected randomly at 72 h after injection to check the gene knockdown by qPCR as described above. All RNAi experiments were carried out at least three times with independently injected head lice (8–22 females each).

For scanning electron microscopy, mature eggs dissected from the uterus or freshly collected eggs laid from *LNSP1*-knockdown or control females were fixed in Karnovsky’s fixative overnight at 4 °C. After washing in 50 mM sodium cacodylate buffer, the eggs were post-fixed using 1% osmium tetroxide in 100 mM sodium cacodylate buffer for 1 h at 4 °C and then dehydrated in a series of 30–100% ethanol baths. The eggs in 100% ethanol were dried in a critical point dryer (EM CPD300, Leica, Austria). Samples were sputter-coated with platinum (EM ACE200, Leica) and analyzed using a field-emission scanning electron microscope (SUPRA 55VP, Carl Zeiss, Germany).

### Inhibition of TG by GGsTop

To inhibit the function of TG in females, 2.3 µg of GGsTop (Tocris Bioscience, Ellisville, MO, USA; 100 mM in 1× PBS and 69 nl/louse) was injected into 5-day-old females as described above (See RNAi section). 1× PBS without GGsTop was injected as control. Injected females were returned to hair tufts with the same number of un-injected males in in vitro rearing system to blood feed. The number of eggs laid during 0 to 24 h after injection were counted and the hatchability of the eggs determined. In parallel, approximately half of injected females were collected randomly at 72 h after injection in order to check the level of gene knockdown by qPCR as described above. All experiments were carried out at least three times with independently injected females (11–18 females each).

To determine the effect of TG inhibitor on the nit sheath solidification after oviposition, human hair was used instead of synthetic hair (wig) for the rearing system. A broom-shaped square hair tuft (25 × 25 mm, 51 strands of human hair) was soaked in 100 mM of GGsTop in 0.1% Triton X-100 in a glass chamber (26 × 26 × 0.17 mm) for 2 h and air-dried for 30 min. The treated hair tuft was placed upright in a feeding chamber. Five 5-day-old females and males were placed onto the hair tuft and allowed to mate and lay eggs for 24 h.

### Statistical analysis

All statistical analyses were performed using GraphPad Prism (version 6, GraphPad Software Inc., San Diego, CA, USA). Means and standard deviations were calculated for each data set and statistical significance was determined by ANOVA followed by Tukey’s post hoc tests and Student’s *t*-tests.

## Results

### Transcriptome profiling of female accessory glands plus uterus

**Fig. 1:**
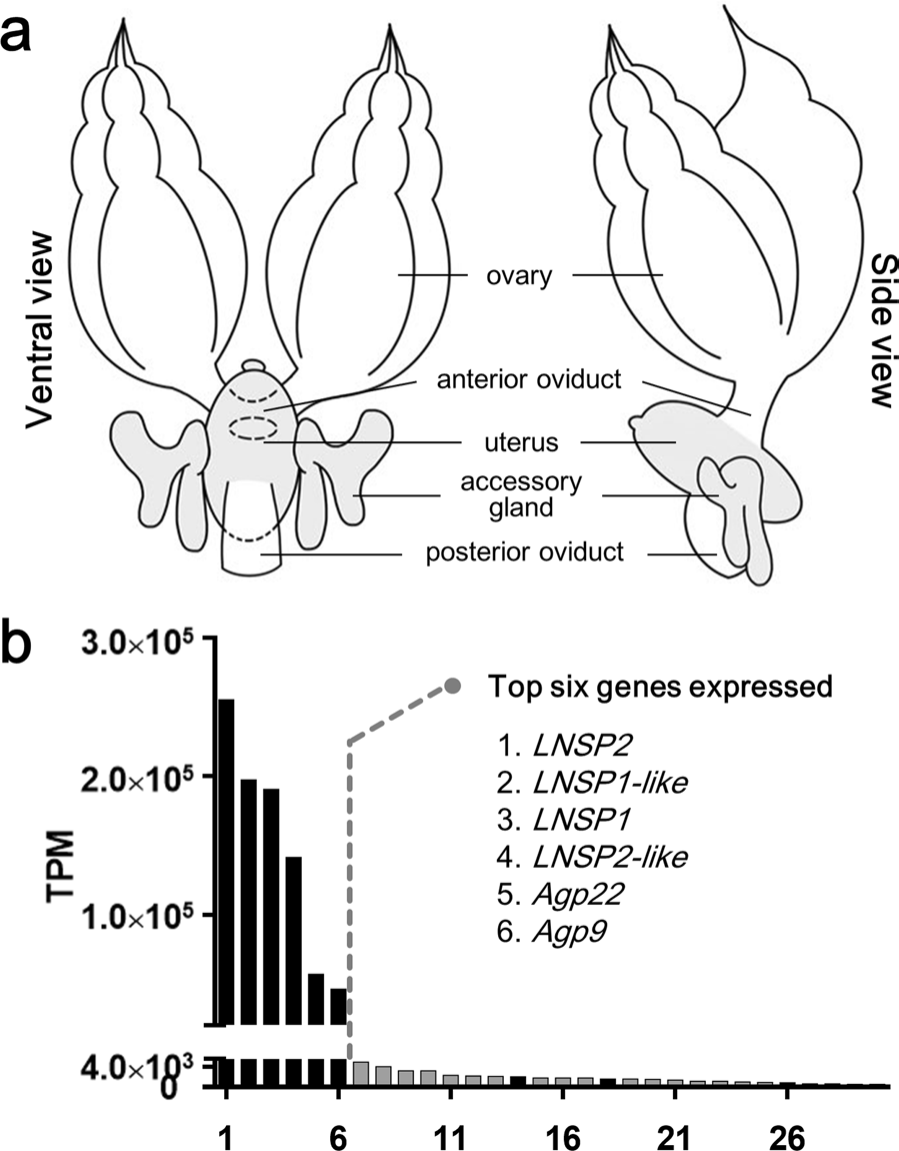
Transcription profiles of the top 30 highly expressed genes in the accessory glands plus uterus. **a** Female reproductive system of human head louse. Tissues (accessory glands plus uterus) used for transcriptome analysis are marked in gray. **b** Transcripts per million (TPM) values of the top 30 genes annotated in the transcriptome of accessory glands plus uterus of 5-day-old head lice. The transcripts of the top six genes occupy 85.7% of whole transcripts expressed in the head louse accessory glands plus uterus. Black or gray bars indicate genes with or without signal peptide sequence, respectively

To identify genes encoding secreted protein components in the accessory gland, the transcriptome of the dissected accessory glands plus uterus from 5-day-old head louse females was analyzed (Fig. 1). Among 8,753 genes annotated, 768 genes were determined to have signal sequences. DEG profiling between accessory gland plus uterus versus ovary (as a control organ) and 0-day old female (at the emergence day) versus 5-day old gravid female revealed that 109 genes were more specifically expressed (more than 10-fold compared to those in ovaries) and that transcripts of only 30 genes occupied 90% of whole expressed transcripts in the accessory gland plus uterus of 5-day old females (Additional file 2: Table S2). The top four genes (*LNSP2*, *LNSP1-like*, *LNSP1* and *LNSP2-like* in order of expression) exhibited extremely high expression levels, and were responsible for 75.4% of the whole transcripts expressed in female accessory glands. In addition, the following two hypothetical genes, named *Agp 22* and *Agp9,* occupied 10.3% of the whole transcripts (Fig. 1b). Therefore, these six genes were presumed to encode the major structural components of the nit sheath. It is still unclear, however, whether *LNSP1*-*like* and *LNSP2*-*like* have interchangeable roles with their intact parental genes or have distinct functions. Nevertheless, the similar combined transcription levels between *LNSP1* plus *LNSP1*-like (387,915 TPM) and *LNSP2* plus *LNSP2-like* (396,387 TPM) and the similarity of gene structures in LNSP1/2 and LNSP1/2-like suggest that both LNSP1-like and LNSP2-like proteins function interchangeably with their respective parental protein. In addition to the putative structural component genes, other genes encoding secretory proteins with putative catalytic or physiological functions included *TG*, *PSI*, defensin 1 and defensin 2 (Additional file 2: Table S2). No genes with allergenic properties were identified from the transcriptomes of accessory gland plus uterus.

**Fig. 2:**
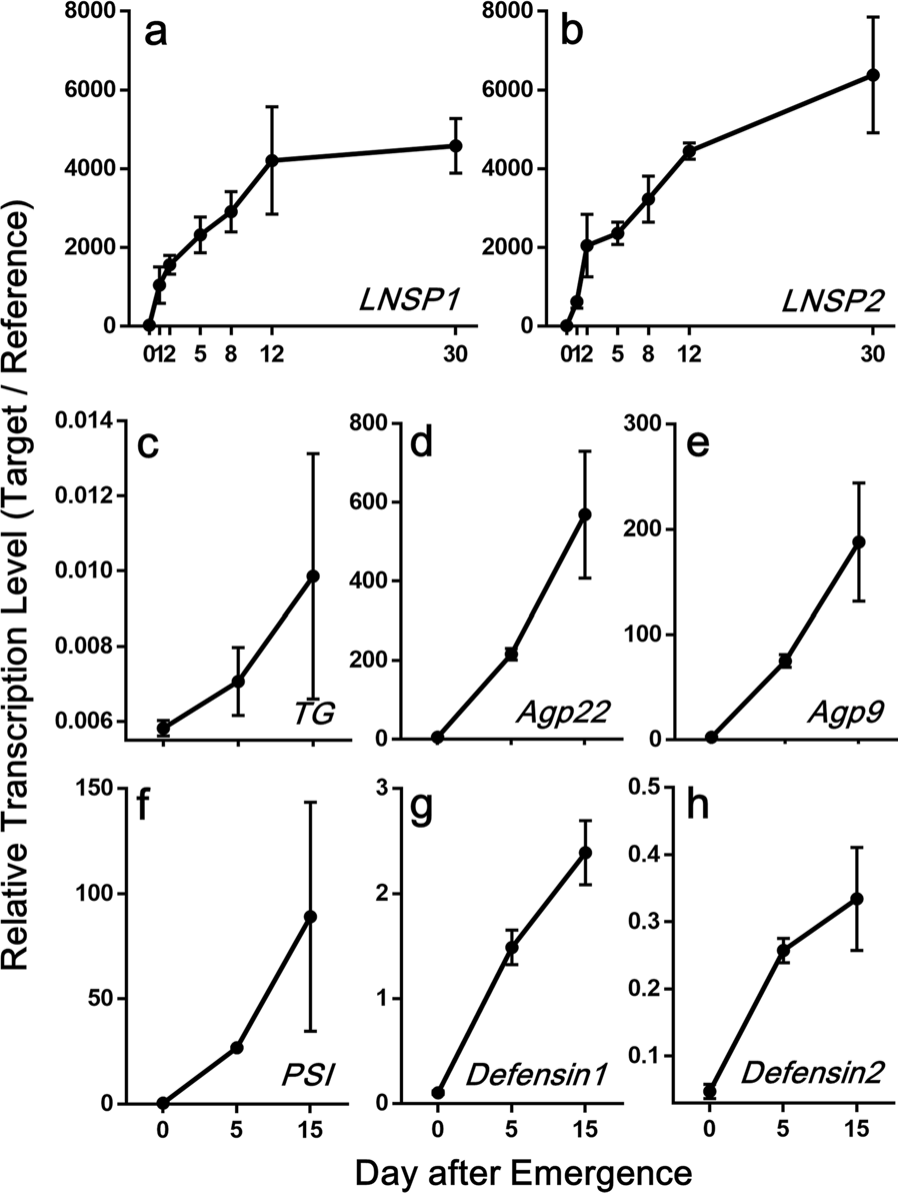
Temporal transcription profiles of major genes in accessory glands plus uterus. Relative transcription levels of **a** *LNSP1*, **b** *LNSP2*, **c** *TG* and **d**–**h** five other major genes in accessory glands plus uterus of female head lice of different ages. Relative transcription levels were normalized by *RpL13A* as a reference gene. Error bars indicate standard deviation

Temporal transcription profiling, as determined by qPCR, confirmed that the transcription levels of all major accessory gland genes increased over time in an age-dependent manner (Fig. 2). In addition, qPCR results using three different tissues (accessory gland plus uterus, ovary and alimentary tract) as templates revealed that all the genes were exclusively expressed in the accessory gland plus oviduct except for *TG* and *PSI* (Additional file 3: Fig. S1). *TG* showed the highest expression pattern in the alimentary tract, followed by the accessory gland plus uterus (Additional file 3: Fig. S1), suggesting its diverse roles in these tissues.

Spatial transcriptional profiling of defensin 1, defensin 2, *Agp22*, *Agp9* and *PSI* genes in separated accessory glands and uterus revealed that both defensin 1 and defensin 2 were 6.3- and 1.8-fold more transcribed in the uterus, respectively, suggesting their protective role against pathogens inside the uterus (Additional file 4: Fig. S2). Transcription levels of *Agp22* and *Agp9* were not significantly different between the two tissues, whereas *PSI* showed a significantly higher transcription level in the accessory glands.

### Functional characterization of several nit sheath proteins via RNAi

#### Knockdown of LNSP1 and LNSP2

In order to investigate the physiological functions of LNSP1 and LNSP2, the corresponding genes were knocked down singly or together. *LNSP1* was 57% (*t*-test, *t*_(4)_ = 6.608, *P* = 0.0053) knocked down by injection of *LNSP1* dsRNA without affecting the transcription of *LNSP2* (Additional file 5: Fig. S3). *LNSP2* was also specifically knocked down (46%; *t*-test, *t*_(4)_ = 3.674, *P* = 0.0213) by injection of *LNSP2* dsRNA without non-specific suppression of LNSP1 transcription (Additional file 5: Fig. S3). Injection of both *LNSP1* and *LNSP2* dsRNAs resulted in 33% (*t*-test, *t*_(4)_ = 5.609, *P* = 0.005) and 29% (*t*-test, *t*_(4)_ = 7.207, *P* = 0.002) knockdown, respectively (Additional file 5: Fig. S3).

**Fig. 3:**
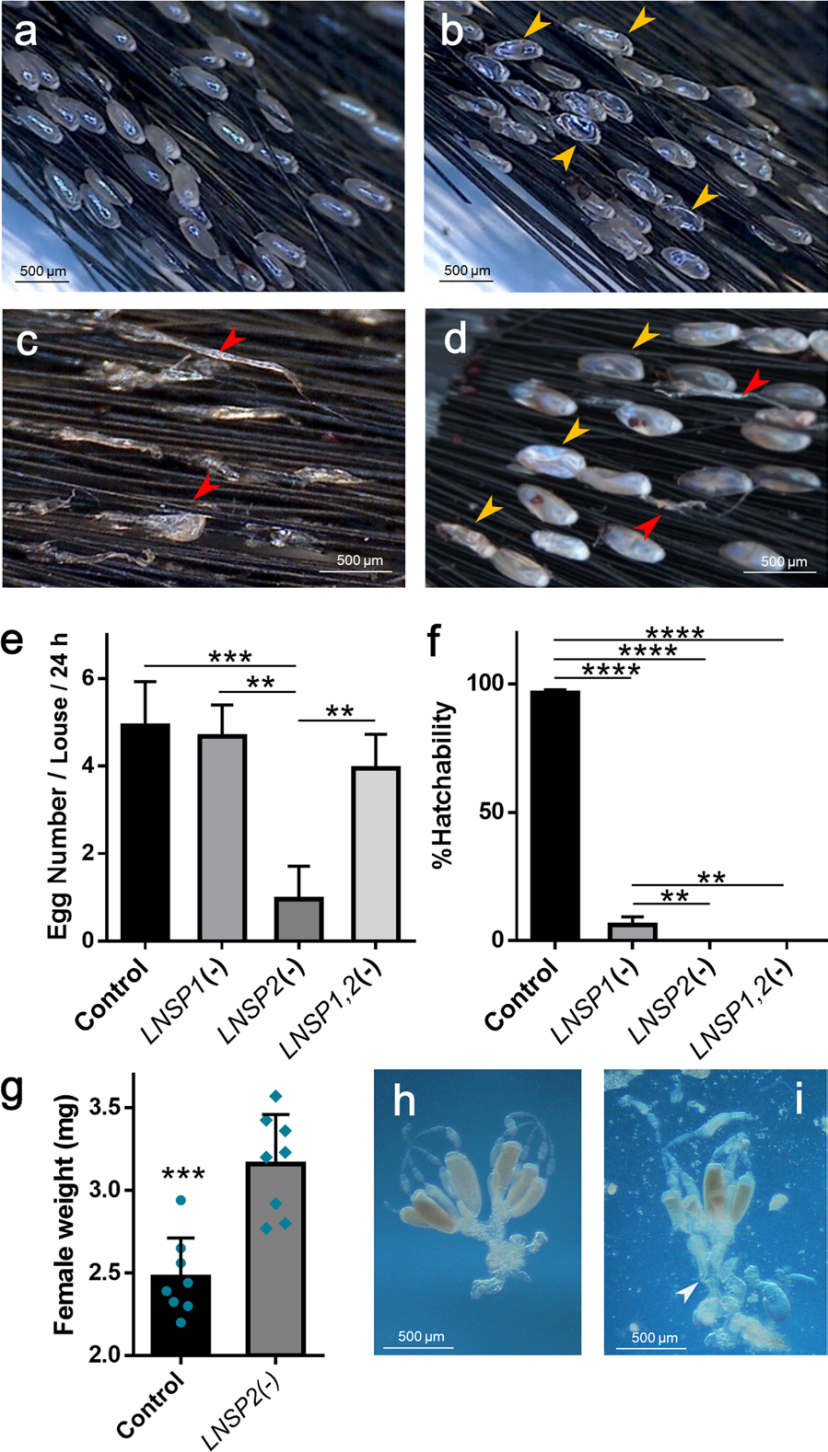
Effects of RNAi-based knockdown of *LNSP1* or *LNSP2* on oviposition. Representative images of the eggs from **a** control, **b** *LNSP1*-knockdown, **c** *LNSP2*-knockdown and **d** *LNSP1*- and *LNSP2*-double knockdown females. Individual dsRNA was injected singly or in combination into the ventral side of an abdomen of a female louse. The eggs were collected on hair tufts for 24 h from 48 to 72 h post-injection. Yellow arrowheads in panels B and D indicate the desiccated dead eggs, and red arrowheads in panels C and D indicate the solidified glue-like material. **e** The number of eggs and **f** %hatchability of eggs laid from the control or *LNSP*-knockdown females during the period 60–84 h post-injection. The hatchability of eggs from the *LNSP2-*knockdown or double-knockdown lice was zero, thus not seen in the graph. **g** Comparison of body weight between the control and *LNSP2*-knockdown females. A reproductive system **h** from a control female and **i** from a *LNSP2*- knockdown female. A white arrowhead in panel I indicates an opening of ruptured uterus. Significant differences were tested using one-way ANOVA (**e**, **f**) or Student’s *t*-test (**g**) (**P < 0.01; ***P < 0.001; ****P < 0.0001). Error bars indicate standard deviations

When *LNSP1* was knocked down, most eggs became shriveled and desiccated (Fig. 3b), which began to appear from 24 h post-injection (Additional file 6: Fig. S4), compared to the control eggs (Fig. 3a). Scanning electron microscopic observations revealed that the surface microstructures of eggs with *LNSP1* knocked down were altered by increased roughness compared to that of control eggs (Additional file 7: Fig. S5). The overall egg number produced from a single female was not significantly affected (ANOVA, *F*_(3,10)_ = 14.73, *P* = 0.7296) but hatchability was dramatically reduced (93.6%; ANOVA, *F*_(3,8)_ = 2382, *P* < 0.0001) as the eggs were severely dehydrated (Fig. 3e, f). Thus, LNSP1 appears to function in desiccation resistance, thereby enhancing the survival of embryo. Nevertheless, no apparent impairment was observed in the gluing function of the nit sheath.

When *LNSP2* transcription was suppressed by RNAi, amorphous glue-like materials were observed to be secreted over hair (Fig. 3c), and overall egg number per female was significantly reduced (80.5%; ANOVA, *F*_(3,10)_ = 14.73, *P* = 0.0011; Fig. 3e). None of the laid eggs hatched out following *LNSP2* knockdown (Fig. 3f). Since *LNSP2*-knockdown females hardly laid any eggs, their body weight significantly increased compared to control females (*t*-test, *t*_(14)_ = 5.078, *P* = 0.0002; Fig. 3g). Unlike the control females (Fig. 3h), dissections of gravid females with LNSP2 knocked down clearly showed ruptured uteri, disintegrated ovaries and enlarged accessory glands (Fig. 3i), suggesting that oviposition is blocked when LNSP2 is not sufficient. Taken together, the nit sheath did not form properly without LNSP2, which appeared to be the main reason for oviposition failure.

Double knockdown of both *LNSP1* and *LNSP2* resulted in the partial restoration of egg production along with the glue-like materials secreted over hair, but the shape of produced eggs was similar to the shriveled eggs with *LNSP1* knocked down (Fig. 3d, e), indicating their susceptibility to desiccation. The egg hatchability was zero, as in the case of *LNSP2* knockdown (Fig. 3f). Overall, the eggs with double knockdown of *LNSP1* and *LNSP2* exhibited mixed characteristics of each single knockdown.

#### Knockdown of other genes

Knockdown of *Agp22*, *Agp9* or *PSI* did not induce any apparent changes in egg number, shape or viability (Additional file 8: Fig. S6), indicating that they may not be as crucial as LNSP1 or LNSP2 in preserving egg viability.

### Elucidation of TG-mediated cross-linking of the nit sheath

**Fig. 4:**
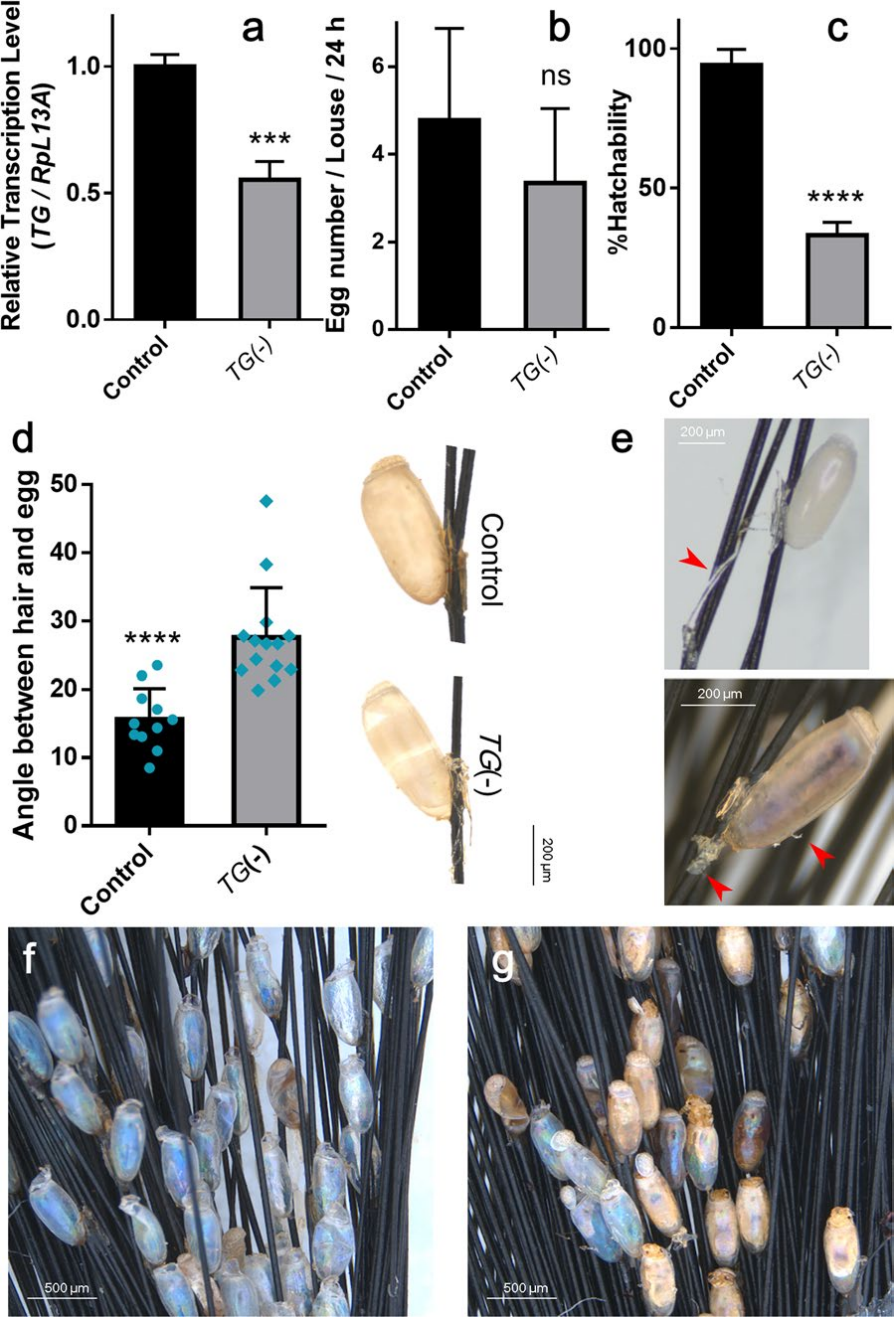
Effects of RNAi-based knockdown of *TG* on oviposition. *TG* dsRNA was injected into the ventral side of an abdomen of a female louse. **a** Relative transcription levels of *TG* at 72 h post-dsRNA injection. **b** The number of eggs and **c** %hatchability of eggs laid from the control or *TG*-knockdown females during 60 ~ 84 h post-dsRNA injection. **d** Comparison of angles between a hair shaft and an egg from the control or *TG*-knockdown females. **e** Thread-like or amorphous glue particles (red arrowheads) found on hair or egg surface following oviposition of *TG*-knockdown females. **f** Empty eggshells from control females at 7 days post-oviposition. All the first instars hatched out. **g** Eggs from *TG*-knockdown females at 7 days post-oviposition, in which stillborn first instars were found inside eggshells. The asterisk marks indicate the statistically significant mean values as judged by Student’s *t*-test (ns, not significant; ***P < 0.001; ****P < 0.0001). Error bars indicate standard deviation

Since the genome-wide survey of possible protein cross-linking systems indicated that TG-mediated cross-linking is the only feasible option, RNAi-based functional characterization of TG was performed. Injection of *TG* dsRNA significantly suppressed *TG* transcription (45%; *t-*test, *t*_(4)_ = 9.106, *P* = 0.0008; Fig. 4a). Knockdown of *TG* via RNAi resulted in reduced egg number although the difference was statistically insignificant (*t*-test, *t*_(5)_ = 1.004, *P* = 0.36; Fig. 4b). Laid eggs appeared normal in shape but the angle between egg and hair was significantly increased (27.6 ± 7.3° compared to 15.7 ± 4.5° in control; *t*-test, *t*_(23)_ = 4.758, *P* < 0.0001; Fig. 4d) and glue particles were observed at the bottom side of eggs (Fig. 4e), suggesting slowed solidification of nit sheath gel following *TG* knockdown. The hatchability was dramatically reduced (64.8%; *t*-test, *t*_(6)_ = 16.07, *P* < 0.0001; Fig. 4c), which was mostly related to the failure of hatching out from eggshells (Fig. 4g) unlike the control eggs (Fig. 4f). Nevertheless, no apparent abnormality in biology (lifespan, viability, etc.) was observed in the females with *TG* knockdown.

**Fig. 5:**
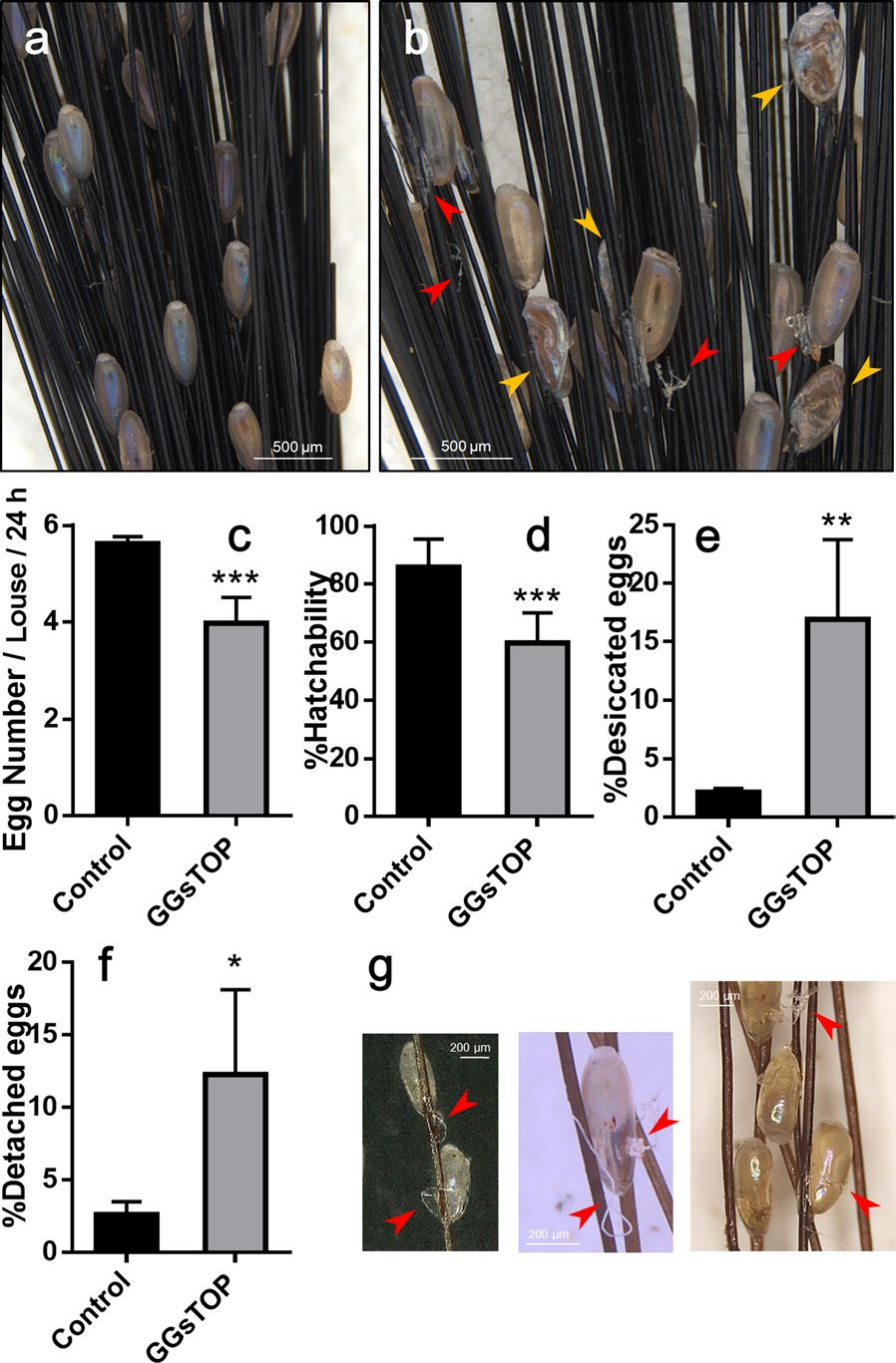
Effects of GGsTop, a *TG* inhibitor, on oviposition. GGsTop was injected into the ventral side of an abdomen of a female louse or coated over hair. Representative images of the eggs from **a** the control and **b** GGsTop-injected females. **c** The number of eggs, **d** %hatchability of eggs, **e** %desiccated eggs and **f** %detached eggs from hairs laid from the control or GGsTop-injected females during 24 h post-treatment. **g** Representative images of eggs laid on GGsTop-treated hair. Yellow arrowheads in panel B indicate the shriveled dead eggs whereas red arrowheads in panels **b** and **g** indicate solidified glue-like material. The asterisk marks indicate the statistically significant mean values as judged by Student’s *t*-test (*P < 0.05; **P < 0.01; ***P < 0.001). Error bars indicate standard deviation

To confirm the role of TG in cross-linking of the nit sheath, TG inhibitors were applied via injection or hair coating. Iodoacetamide, a well-known potent and irreversible TG inhibitor, was too toxic to females. Since the toxic effects of iodoacetamide were exerted within a very narrow window of doses, regardless of treatment routes (hair coating vs. injection), no meaningful dose–response was deduced (Additional file 9: Table S2). Therefore, GGsTop was used as a surrogate inhibitor because it is known as an irreversible inhibitor of gamma-glutamyltransferase (EC 2.3.2.2), which is closely related with TG (EC 2.3.2.13), and because no genes encoding the gamma-glutamyltransferase were annotated from the human louse genome. Injection of GGsTop into gravid females resulted in similar responses as observed in the *TG* knockdown (Fig. 5). Injection of GGsTop caused a decreased total number of eggs laid (1.4-fold, *t*-test, *t*_(6)_ = 5.999, *P* = 0.0010; Fig. 5c), a decrease in their hatchability (1.4-fold, *t*-test, *t*_(14)_ = 5.189, *P* = 0.0001; Fig. 5d) and an increased number of desiccated eggs found on the hair (7.9-fold, *t*_(6)_ = 4.327, *P* = 0.0049; Fig. 5e). In addition, 12.3% of total eggs laid by GGsTop-injected females were detached from hairs (Fig. 5f). When females laid eggs on the hair tufts coated with GGsTop, thread- or plate-shaped gel materials, produced by oviposition behavior, were observed around the eggs (Fig. 5g), suggesting that solidification of the nit sheath was slowed at the post-oviposition stage. However, no reduction in egg hatchability or structural abnormality of eggs were observed, indicating that the egg coating and cross-linking of nit sheath gel inside the uterus during the pre-oviposition stage is sufficient to ensure egg viability. Taken together, these findings suggest that GGsTop inhibited TG activity, and thus suppressed the cross-linking mediated by TG, impairing the egg viability.

## Discussion

### Accessory gland secreted proteins

Transcriptome analysis of the female accessory gland enabled the identification of all four protein components that were previously reported (LNSP1, LNSP2, Agp22 and Agp9) based on their secretory nature and extremely high transcription levels. In addition to these putative structural components of the nit sheath, several proteins, such as TG, PSI and two defensins, were identified to provide some relevant functions required for nit sheath formation and oviposition. All the above-mentioned genes exhibited the age-dependent and accessory gland-specific (except for TG and defensins) expression patterns, implying their specialized roles in egg production and oviposition. The age-dependent expression pattern of LNSP was also observed in virgin females that are able to lay unfertilized eggs, suggesting the increase of transcription levels is not triggered by mating.

It is intriguing that both defensin 1 and defensin 2, which are the only antimicrobial peptides of human lice [12], were secreted from the oviduct perhaps to protect eggs and reproductive organs from microbial invasion. It remains to be elucidated, however, whether these defensins are coated over or incorporated into the nit sheath as an extended protection process or simply function as a defense system against microbial infection inside the reproductive organ. In the case of human lice, it is currently unknown whether serosal membrane is present in the egg and is actively involved in immune response.

*Candidatus* Riesia pediculicola, the primary endosymbiont of the head louse, is located inside the paired oviduct, called ovarial ampullae, of mature females and migrates to the posterior poles of oocytes for vertical transmission [13]. Considering that the immune system of head lice might control the titer of *Ca*. R. pediculicola [14] and the secreted defensin 1 and defensin 2 can directly interact with the extracellular *Ca*. R. pediculicola during migration in the oviducts, defensins can regulate their density and viability in the new embryo by likely preventing the initial colonization. Since defensin 2 is also transcribed in the ovary (Additional file 3: Fig. S1), it appears to play a role in regulating the density of primary endosymbiont in the ovarial ampullae and its subsequent moving toward oocytes.

### Roles of LNSP1 and LNSP2

Knockdown of LNSP1 did not affect the average number of eggs produced from a single female but significantly reduced hatchability due, in part, to a severe level of desiccation in eggs (Fig. 3e, f). These findings demonstrate that LNSP1 plays a critical role in preserving water in the developing egg and thus maintaining egg viability.

Based on the observation of a glue-like material secreted over the hair tuft following *LNSP2* knockdown, it appears that LNSP1 by itself cannot be assembled as nit sheath. This finding is in contrast to the effect of *LNSP1* knockdown, in which the nit sheath was formed with LNSP2 alone despite having a defective function. This result further suggests that LNSP2 may serve as the basic platform to form the nit sheath, without which it cannot be assembled. From the finding that mature eggs lacking LNSP2 were stuck inside the uterus, leading to disintegration of the ovary, enlargement of the accessory gland and eventual failure of oviposition, it can be speculated that LNSP2 likely has an additional function of lubrication. The secretory material from female accessory glands is also known to function as lubricant for egg passage [15]. This evidence indicates that LNSP2 may serve as the basic platform for the nit sheath assembly and also provides a lubricating function.

Considering that a functional nit sheath is only formed when both LNSP1 and LNSP2 are equally present, it appears that the stoichiometry between LNSP1 and LNSP2 is crucial and the ratio needs to be balanced to produce a functional nit sheath. If the balance between the two is impaired, as observed in the case of single knockdown, nit sheath formation is blocked (see LNSP2 knockdown) or nit sheath with defective function (see LNSP1 knockdown) is formed. Based on the combined transcription levels of *LNSP1*/*LNSP1-like* and *LNSP2*/*LNSP2-like*, the required stoichiometry between LNSP1 and LNSP2 appears to be 1:1.

Interestingly, when both *LNSP1* and *LNSP2* were knocked down, egg production was partially restored, which is in contrast to the significantly reduced egg production following single knockdown of *LNSP2*. In the case of double knockdown of both *LNSP1* and *LNSP2*, a balanced cross-linking may be feasible between LNSP1 and LNSP2, which allows the formation of minimum layers of nit sheath having lubricating function, thereby barely allowing oviposition despite their reduced quantity due to RNAi. However, the overall amounts of LNSP1 and LNSP2 appear insufficient to meet the critical level necessary for ensuring a functional nit sheath, thereby impairing egg viability as evidenced by the lack of egg hatch. Therefore, the nit sheath may be regarded as an integral part of the eggshell considering its critical roles in maintaining egg viability. In addition to their function as glue for attaching eggs to hair, LNSP1 and LNSP2 fulfill additional roles, including water preservation, that are essential for maintaining viability of eggs. The additional putative structural components, such as Agp22 and Agp9, did not affect egg number, shape or viability when knocked down (Additional file 8: Fig. S6), suggesting their relatively insignificant role compared to LNSP1 or LNSP2 in preserving egg viability.

### TG-mediated cross-linking of the nit sheath

TG was previously suggested as the most feasible enzyme that can mediate the cross-linking of LNSP1 and LNSP2 [9]. In fact, RNAi-based knockdown of *TG* impaired normal egg production, most likely due to defect cross-linking of LNSP1 and LNSP2. The dramatically reduced hatchability was mostly attributed to the failure of hatching from the nit sheath (Fig. 4g), suggesting that the extent of cross-linking suppression by *TG* knockdown was not rapid enough to induce early embryonic death, but resulted in incomplete embryo development. If assuming that a normal nit sheath plays a critical role in protecting the primary endosymbiont *Ca.* R. pediculicola associated with new oocytes from the host immune system (i.e., defensin 1 and defensin 2 in the oviduct), the defective nit sheath caused by TG knockdown might impair the successful initial colonization of *Ca.* R. pediculicola and/or affect its viability or density in the egg, thereby resulting in the incomplete embryo development and eventual hatching failure.

Similar to the *TG* knockdown, administration of GGsTop, a TG inhibitor, via either hair coating or injection into gravid females, resulted in similar consequences. These findings demonstrate that TG is involved in cross-linking of LNSP1 and LNSP2. Taken together, the TG-mediated cross-linking mechanism can be employed as a therapeutic target to control human louse eggs, and any topically applied TG inhibitors can be exploited as potential ovicidal agents.

TG catalyzes the formation of amide bonds between ε-amino group of a Lys side chain and γ-carboxamide group of a Gln side chain in proteins, resulting in insoluble cross-linked protein aggregates [16, 17]. The cross-linked insoluble protein aggregates are highly resistant to mechanical challenge and proteases degradation, and are essential in the formation of stable structures in several tissues and processes, including skin, hair, blood clotting and wound healing [16]. The nit sheath of the head louse was also extremely resistant to various chemical treatments [9], providing a rigid and stable structure as a protective covering for eggs. Considering the water-preserving capability, affinity to human hair (keratin) and structural rigidity of the nit sheath, the nit sheath gel composed LNSP1 and LNSP2, if properly regulated by TG-mediated cross-linking, could be exploited as materials for medical and cosmetic uses, including the application as wound coagulant and hydrogel for skin protection [18]. The coevolutionary history of humans and human head lice during their long host-parasite relationships suggests no or little allergic properties associated with LNSP1 and LNSP2, rendering them more suitable for human use.

The patterns of intramolecular or intermolecular cross-linking of LNSP1 and LNSP2 could not be deduced in this study. Both LNSP1 and LNSP2 have similar structures, are composed of three domains of N-terminal polyQA, middle polyGA and C-terminal polyQ, and contain many Gln residues [9] along with 12–13 Lys residues. Thus, it is possible that both intramolecular cross-linking within each of the LNSPs and intermolecular cross-linking between LNSP1 and LNSP2 or with other, yet unidentified molecules would form.

Although TG was most abundantly expressed in the alimentary tract, either knockdown of *TG* or treatment with GGsTop did not induce any apparent negative impacts on female biology. One group within the TG family, called tissue TG (tTG), is expressed in the cytosol of many different organs in humans, including the small intestine [19] and the intracellular TG appears to play a critical role in apoptosis [20]. This finding suggests that TG expressed in the alimentary tract is likely involved in non-acute cellular processes such as apoptosis, thereby evoking little acute negative impacts, when knocked down.

## Conclusions

Both LNSP1 and LNSP2 are major constituents of the nit sheath that forms a rigid and stable egg covering. Along with functioning as glue for attaching eggs to human hair and as an egg covering for mechanical protection, LNSP1 and LNSP2 play critical roles in water preservation that are essential for ensuring normal embryogenesis. Due to its water-preserving capability, gluing property and structural rigidity, the nit sheath gel with regulated TG-mediated cross-linking could be exploited as materials for medical and cosmetic uses. The cross-linking mechanism mediated by TG can be employed as a therapeutic target to control human louse eggs, and any topically applied TG inhibitors with appropriate water/octanol coefficient allowing it to penetrate the louse body, can be exploited as a novel ovicidal agent or a chemical means facilitating the physical removal of nits. Moreover, the transcriptomic analysis of female accessory gland as performed in this study should facilitate the identification of structural and functional components from any egg sheath of parasitic arthropods, and can be employed as a routine tool for searching for novel peptide/protein components with useful functions.

## Supplementary Information

**Additional file 1: Table S1.** Primers used in this study.

**Additional file 2: Table S2.** List of genes abundantly expressed in the accessory glands plus uterus of female head lice. Top 30 abundantly expressed genes are listed in no. 1–30, whereas three putative functionally important genes are listed in no. 31–33.

**Additional file 3: Figure S1.** Spatial transcription profiles of eight major genes in three different female organs. All transcription levels were normalized by those in accessory gland. The transcription levels of *LNSP1*, *LNSP2*, *Agp22* in alimentary tract and of defensin 1 in ovary were very low (below 0.0003), thus not seen in the graph. Error bars indicate standard deviation.

**Additional file 4: Figure S2.** Comparisons of spatial transcription profiles of several genes between the accessory gland and the uterus. Relative transcription levels in 5-day-old female lice were normalized by *RpL13A* as a reference gene. The asterisks indicate the statistically significant mean values as judged by Student’s *t*-test (**P < 0.01). Error bars indicate standard deviation.

**Additional file 5: Figure S3.** Relative transcription levels of LNSP1 and LNSP2 following RNAi-based knockdown. RNAi was conducted by injecting dsRNA of *LNSP1* (**a**), *LNSP2* (**b**) or (**c**) *LNSP1* plus *LNSP2* (double RNAi), and transcription levels were determined at 72 h post-injection*. LNSP1* was specifically knocked down by injection of *LNSP1* dsRNA without non-specific suppression of *LNSP2* transcription, and vice versa. The asterisks indicate the statistically significant mean values compared to control as judged by Student’s *t*-test (*P < 0.05; **P < 0.01; ***P < 0.001). Error bars indicate standard deviation.

**Additional file 6: Figure S4.** Representative images of head louse eggs observed by stereomicroscopy (×20). Eggs were obtained from the control (**a**) and *LNSP1*-knockdown (**b**) females. The eggs were collected for 24 h after dsRNA injection. Red arrows in panel B indicate the eggs with shriveled surface.

**Additional file 7: Figure S5.** Representative images of head louse egg surface observed by scanning electron microscopy (10,000×). **a** An egg without nit sheath dissected from anterior oviduct. **b** An egg with regular nit sheath from a control head louse. **c**, **d** Typical irregular shapes of surface of eggs from *LNSP1*-knockdown head louse.

**Additional file 8: Figure S6.** Effects of RNAi-based knockdown of *Agp22*, *Agp9* or *PSI* on oviposition. **a** Relative transcription levels of *Agp22*, *Agp9* and *PSI* at 72 h post-dsRNA injection. **b** The number of eggs and **c** %hatchability of eggs laid from control or knockdown females during 60 ~ 84 h after dsRNA injection. Significant differences were tested using Student’s *t*-test (a; ***P < 0.001; ****P < 0.0001) or one-way ANOVA (b, c; ns, non-significant). Error bars indicate standard deviation.

**Additional file 9: Table S3.** Mortality of iodoacetamide (IAA)-injected female head lice.

**Additional file 10: Table S4.** The total numbers of eggs examined and the proportions of affected eggs by various treatments.

## Data Availability

The sequencing data of transcriptome analysis conducted in this study have been submitted to NCBI under BioProject PRJNA694820. The number of eggs and their hatchability are listed in Additional file 10: Table S4.

## Abbreviations

Agp: Accessory gland protein
LNSP: Louse nit sheath protein
PSI: Pacifastin-like serine protease inhibitor
RNAi: RNA interference
TG: Transglutaminase
TPM: Transcripts per million

## References

[1] Resh VH, Cardé RT (2009). Encyclopedia of insects.

[2] Hilker M, Meiners T (2008). Chemoecology of insect eggs and egg deposition.

[3] Clark JM (2009). Determination, mechanism and monitoring of knockdown resistance in permethrin-resistant human head lice, *Pediculus humanus capitis*. J Asia-Pacif Entomol.

[4] Ferris GF. The sucking lice. *Mem Pacif Coast ent Soc* 1951.

[5] Carter DG (1990). Insect egg glue: an investigation of the nature and secretion of insect egg glues, with special reference to the human louse, *Pediculus humanus* and the cabbage white butterfly, *Pieris brassicae*.

[6] Burkhart C, Arbogast J, Smythe P, Burkhart C (1999). Histochemical analysis of the nit of *Pediculus humanus capitis* (Anoplura: Pediculidae). J Med Entomol.

[7] Burkhart CN, Stankiewicz BA,  Pchalek I, Kruge  MA, Burkhart CG (1999). Molecular composition of the louse sheath. J
Parasitol.

[8] Burkhart C. Nit sheath and bacterial symbiotes of the human head louse (*Pediculus humanus capitis*). Master’s thesis, Medical College of Ohio (Toledo, Ohio, 2002); 2002.

[9] Park JK, Han YJ, Lee JH, Joo S-W, Kim JH, Lee SH, Park S (2019). Characterization of the human head louse nit sheath reveals proteins with adhesive property that show no resemblance to known proteins. Sci Rep.

[10] Yoon KS, Strycharz JP, Gao J-R, Takano-Lee M, Edman JD, Clark JM (2006). An improved in vitro rearing system for the human head louse allows the determination of resistance to formulated pediculicides. Pestic Biochem Physiol.

[11] Pfaffl MW (2001). A new mathematical model for relative quantification in real-time RT–PCR. Nucleic Acids Res.

[12] Kim JH, Min JS, Kang JS, Kwon DH, Yoon KS, Strycharz J, Koh YH, Pittendrigh BR, Clark JM, Lee SH (2011). Comparison of the humoral and cellular immune responses between body and head lice following bacterial challenge. Insect Biochem Mol Biol.

[13] Sasaki-Fukatsu K, Koga R, Nikoh N, Yoshizawa K, Kasai S, Mihara M, Kobayashi M, Tomita T, Fukatsu T (2006). Symbiotic bacteria associated with stomach discs of human lice. Appl Environ Microbiol.

[14] Perotti MA, Allen JM, Reed DL, Braig HR (2007). Host-symbiont interactions of the primary endosymbiont of human head and body lice. FASEB J.

[15] Hoffmann KH. Oogenesis and the female reproductive system. In: Insect reproduction. 1995;1–32.

[16] Griffin M, Casadio R, Bergamini CM (2002). Transglutaminases: nature’s biological glues. Biochem J.

[17] Yokoyama K, Nio N, Kikuchi Y (2004). Properties and applications of microbial transglutaminase. Appl Microbiol Biotechnol.

[18] Pourshahrestani S, Zeimaran E, Kadri NA, Mutlu N, Boccaccini AR (2020). Polymeric hydrogel systems as emerging biomaterial platforms to enable hemostasis and wound healing. Adv Healthcare Mater.

[19] Klöck C, DiRaimondo TR, Khosla C (2012). Role of transglutaminase 2 in celiac disease pathogenesis. Semin Immunopathol.

[20] McConkey DJ, Orrenius S (1997). The role of calcium in the regulation of apoptosis. Biochem Biophys Res Commun.

