# Supplementary material for: Characterization of nit sheath protein functions and transglutaminase-mediated cross-linking in the human head louse, Pediculus humanus capitis
Source: Parasit Vectors. 2021 Aug 24;14:425. doi: 10.1186/s13071-021-04914-z (PMC8383413; doi:10.1186/s13071-021-04914-z)
Supplement: Supplementary file 1 — Additional file 1: Table S1. Primers used in this study. [file 13071_2021_4914_MOESM1_ESM.docx]

**Table S1.** Primers used in this study.

| Purpose | Gene | Sequence (5’ to 3’) | | Product size (bp) |
| --- | --- | --- | --- | --- |
| qPCR | *RpL13A* | F^a^  R^b^ | GTTAGGGGAATGCTTCCACAC  GGTCTAAGGCAGAGAACGCT | 142 |
|  | *LNSP1* | F  R | GTCCAATCTGGTGTCTGGCA  CCCACATGTGATGACCTTGA | 106 |
|  | *LNSP2* | F  R | GCTCAAGCTCATGCTCAAGC  ACTCTTCTGCTTCCGACGAC | 110 |
|  | *TG* | F  R | ATGGCCAGAGCTATTTCCGC  CGGTCCATGCAAATGGTGAC | 109 |
|  | *Agp22* | F  R | GTTCAACCCATCGTAGTTGC  GTTCCAGCGTCTTGAACGGA | 122 |
|  | *Agp9* | F  R | ACTTACGGACCTAATGGCGG  TCCGTTGACGCTACTCGTAC | 110 |
|  | *PSI* | F  R | CGAAGAAACTGGTGCTCAAG  TCAAACCGCTGTCTGGACAC | 113 |
|  | *Def1* | F  R | GGGAGAACTTACCTCGGAAA  AGCGGCACAAGCAGAATGAT | 142 |
|  | *Def2* | F  R | TGGAGGAAGATTCAGGAGAGC  GCAACGTCCACCTTTGTAACC | 127 |
| dsRNA | *LNSP1* | F  R | ^c^ TAATACGACTCACTATAGGGACTTTTCGCCGCTGGATTCG  TAATACGACTCACTATAGGGGTTCTTGAACGACGGGGGAA | 538 |
|  | *LNSP2* | F  R | TAATACGACTCACTATAGGGTTCGTACCCCATGCACGGAT  TAATACGACTCACTATAGGGTCCTTTTCCGTAACCGGTGG | 590 |
|  | *TG* | F  R | TAATACGACTCACTATAGGGGCATTTGCCGCTGTCAATGC  TAATACGACTCACTATAGGGGTCCAAATATTCCTCGGGAG | 531 |
|  | *Agp22* | F  R | TAATACGACTCACTATAGGGTGCCTGCATTTATGGCGCCA  TAATACGACTCACTATAGGGACCACCAGATTTTCCGTGAC | 505 |
|  | *Agp9* | F  R | TAATACGACTCACTATAGGGGTTGTTGACATTCCCAAGCG  TAATACGACTCACTATAGGGCCATTGTCGTTCGTTCCGAA | 251 |
|  | *PSI* | F  R | TAATACGACTCACTATAGGGGCACGCACAATCAACGTTTC  TAATACGACTCACTATAGGGGAGTACATCCGGCCAATGAT | 362 |
|  | pQE30 | F  R | TAATACGACTCACTATAGGGAGACATCCATAGTTGCCTGACTC  TAATACGACTCACTATAGGGAGATAACACTGCGGCCAACTTAC | 422 |
