# Supplementary material for: Characterization of nit sheath protein functions and transglutaminase-mediated cross-linking in the human head louse, Pediculus humanus capitis
Source: Parasit Vectors. 2021 Aug 24;14:425. doi: 10.1186/s13071-021-04914-z (PMC8383413; doi:10.1186/s13071-021-04914-z)
Supplement: Supplementary file 2 — Additional file 2: Table S2. List of genes abundantly expressed in the accessory glands plus uterus of female head lice. Top 30 abundantly expressed genes are listed in no. 1–30, whereas three putative functionally important genes are listed in no. 31–33. [file 13071_2021_4914_MOESM2_ESM.docx]

**Table S2.** List of genes abundantly expressed in the accessory glands plus uterus of female head lice. Top 30 abundantly expressed genes are listed in no. 1-30, whereas three putative functionally important genes are listed in no. 31-33.

| No. | Accession No. | Function | TPM |
| --- | --- | --- | --- |
| 1 | XP_002432622.1 | **LNSP2** | 225134 |
| 2 | XP_002428823.1 | LNSP1-like | 197467 |
| 3 | XP_002432620.1 | **LNSP1** | 190448 |
| 4 | XP_002432621.1 | LNSP2-like | 141253 |
| 5 | XP_002428146.1 | **Agp22** | 56887.2 |
| 6 | XP_002432257.1 | **Agp9** | 45982.2 |
| 7 | XP_002425519.1 | conserved hypothetical protein | 4849.8 |
| 8 | XP_002432542.1 | hypothetical protein | 3970.0 |
| 9 | XP_002428583.1 | conserved hypothetical protein | 3197.6 |
| 10 | XP_002432543.1 | conserved hypothetical protein | 3197.6 |
| 11 | XP_002427897.1 | hypothetical protein | 2318.9 |
| 12 | XP_002425521.1 | hypothetical protein | 2150.9 |
| 13 | XP_002430709.1 | stress associated endoplasmic reticulum protein | 2112.9 |
| 14 | XP_002424036.1 | acidic ribosomal protein P1 | 1983.8 |
| 15 | XP_002425917.1 | hypothetical protein | 1821.8 |
| 16 | XP_002427899.1 | hypothetical protein | 1813.5 |
| 17 | XP_002429132.1 | hypothetical protein | 1813.5 |
| 18 | XP_002432611.1 | **Pacifastin-like serine protease inhibitor (PSI)** | 1600.4 |
| 19 | XP_002425849.1 | conserved hypothetical protein | 1559.9 |
| 20 | XP_002429746.1 | 40S ribosomal protein S27 | 1479.4 |
| 21 | XP_002426744.1 | conserved hypothetical protein | 1326.8 |
| 22 | XP_002424793.1 | conserved hypothetical protein | 1144.0 |
| 23 | XP_002427898.1 | hypothetical protein | 1132.6 |
| 24 | XP_002431869.1 | L-ascorbate oxidase | 955.5 |
| 25 | XP_002425887.1 | 40S ribosomal protein S29 | 897.0 |
| 26 | XP_002423790.1 | 60S ribosomal protein L39 | 825.2 |
| 27 | XP_002425950.1 | predicted protein | 657.7 |
| 28 | XP_002426688.1 | actin | 626.6 |
| 29 | XP_002426517.1 | 60S ribosomal protein L27e | 585.1 |
| 30 | XP_002430380.1 | steroidogenic factor | 579.0 |
| 31 | XP_002428138.1 | **Defensin 1** | 168.8 |
| 32 | XP_002432619.1 | **Defensin 2** | 32.6 |
| 33 | XP_002429622.1 | **Transglutaminase (TG)** | 2.29 |
