# Supplementary figures and images for: Characterization of nit sheath protein functions and transglutaminase-mediated cross-linking in the human head louse, Pediculus humanus capitis
Source: Parasit Vectors. 2021 Aug 24;14:425. doi: 10.1186/s13071-021-04914-z (PMC8383413; doi:10.1186/s13071-021-04914-z)

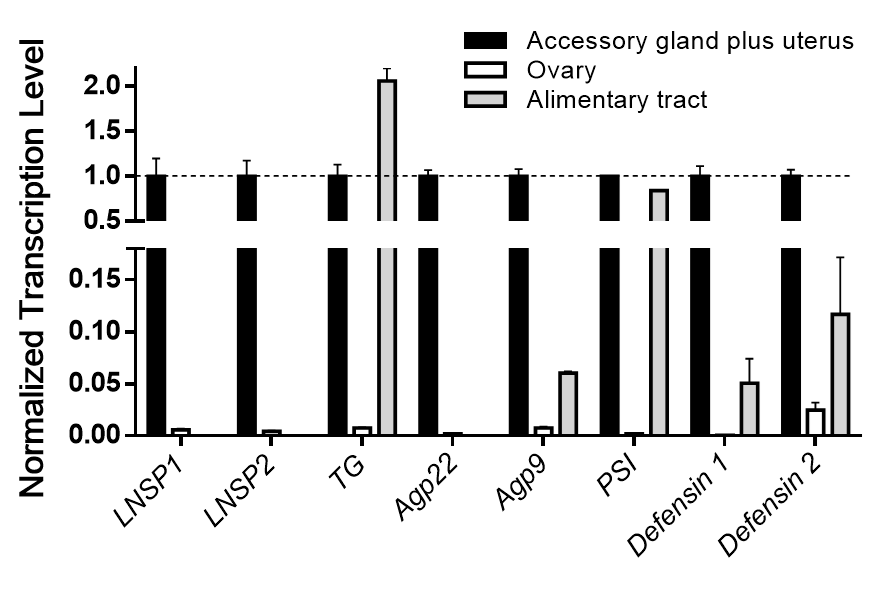

Supplement: Supplementary file 3 — Additional file 3: Figure S1. Spatial transcription profiles of eight major genes in three different female organs. All transcription levels were normalized by those in accessory gland. The transcription levels of LNSP1, LNSP2, Agp22 in alimentary tract and of defensin 1 in ovary were very low (below 0.0003), thus not seen in the graph. Error bars indicate standard deviation. [file 13071_2021_4914_MOESM3_ESM.tif]

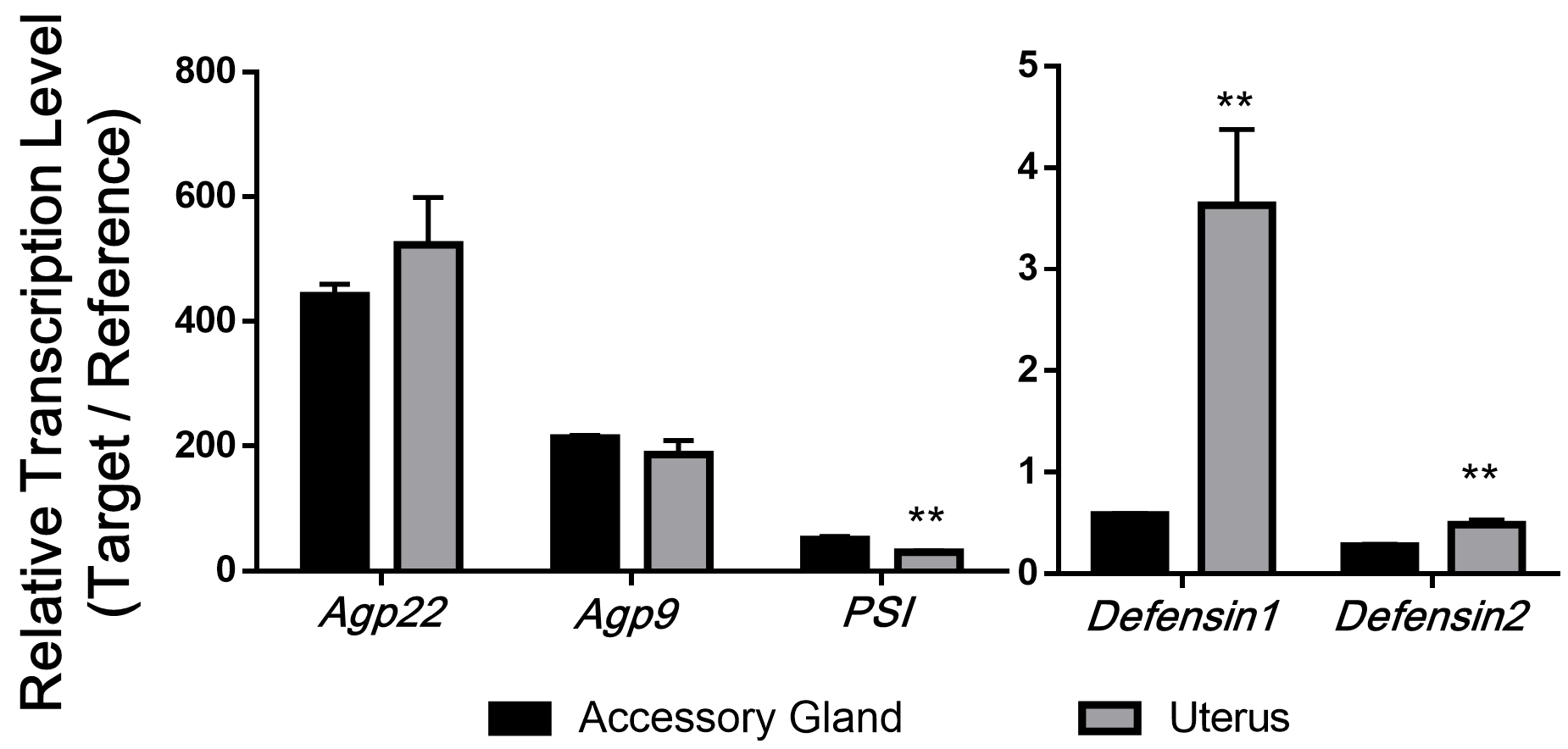

Supplement: Supplementary file 4 — Additional file 4: Figure S2. Comparisons of spatial transcription profiles of several genes between the accessory gland and the uterus. Relative transcription levels in 5-day-old female lice were normalized by RpL13A as a reference gene. The asterisks indicate the statistically significant mean values as judged by Student’s t-test (**P < 0.01). Error bars indicate standard deviation. [file 13071_2021_4914_MOESM4_ESM.tif]

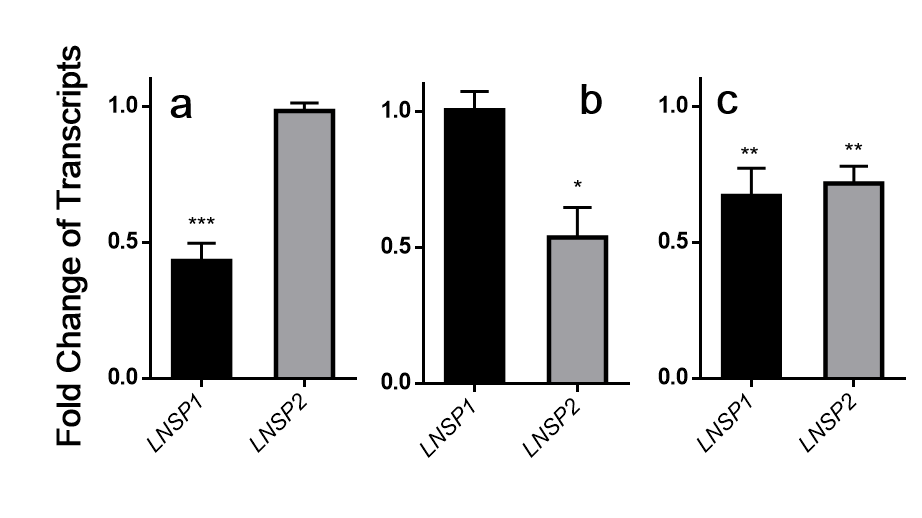

Supplement: Supplementary file 5 — Additional file 5: Figure S3. Relative transcription levels of LNSP1 and LNSP2 following RNAi-based knockdown. RNAi was conducted by injecting dsRNA of LNSP1 (a), LNSP2 (b) or (c) LNSP1 plus LNSP2 (double RNAi), and transcription levels were determined at 72 h post-injection. LNSP1 was specifically knocked down by injection of LNSP1 dsRNA without non-specific suppression of LNSP2 transcription, and vice versa. The asterisks indicate the statistically significant mean values compared to control as judged by Student’s t-test (*P < 0.05; **P < 0.01; ***P < 0.001). Error bars indicate standard deviation. [file 13071_2021_4914_MOESM5_ESM.tif]

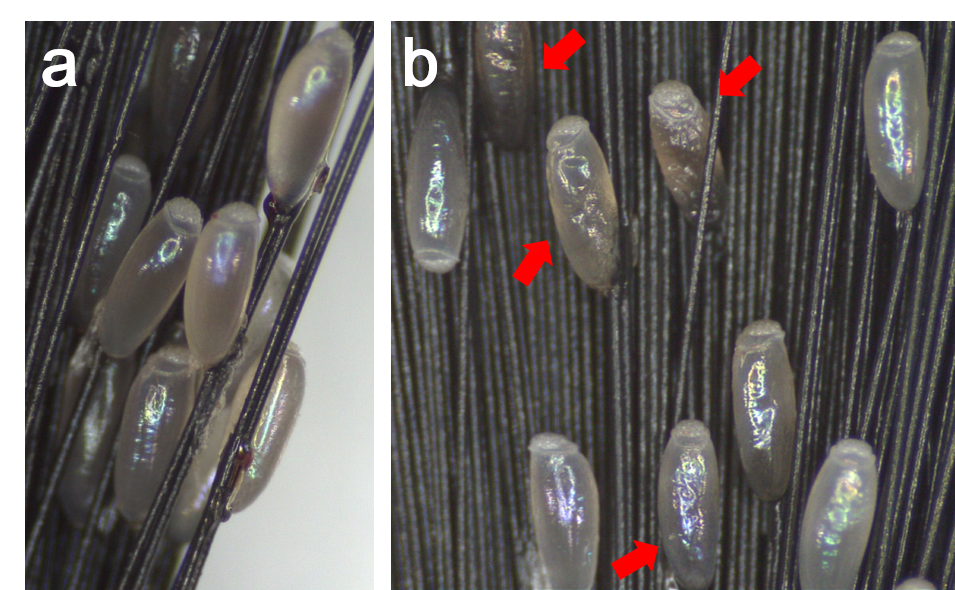

Supplement: Supplementary file 6 — Additional file 6: Figure S4. Representative images of head louse eggs observed by stereomicroscopy (×20). Eggs were obtained from the control (a) and LNSP1-knockdown (b) females. The eggs were collected for 24 h after dsRNA injection. Red arrows in panel B indicate the eggs with shriveled surface. [file 13071_2021_4914_MOESM6_ESM.tif]

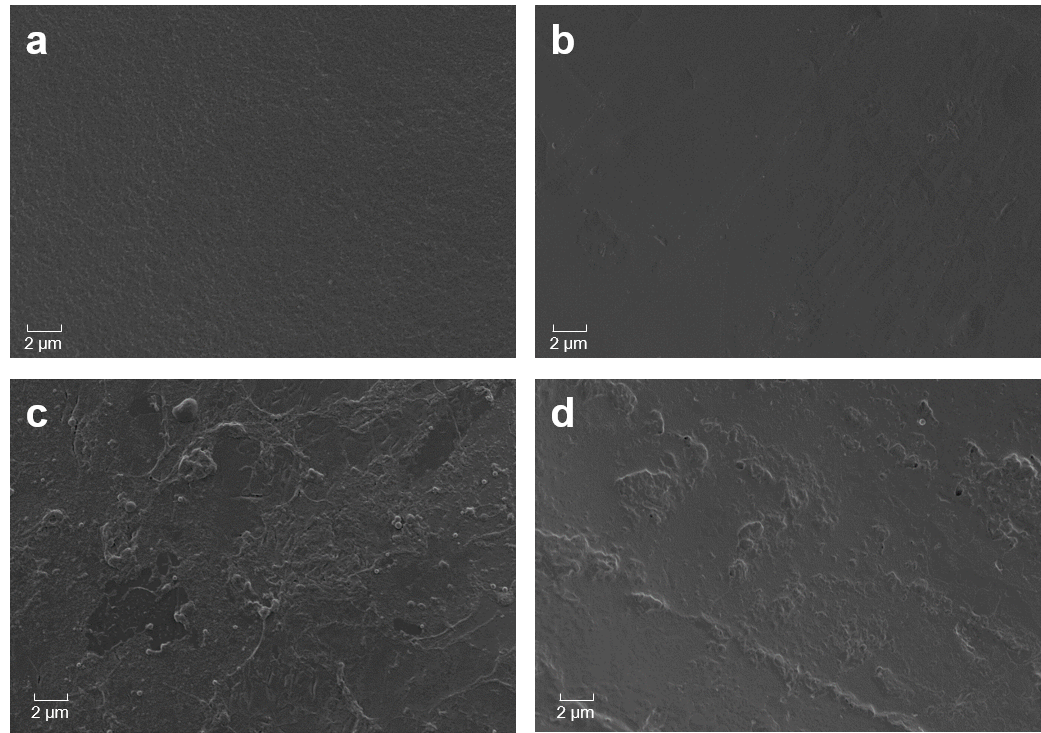

Supplement: Supplementary file 7 — Additional file 7: Figure S5. Representative images of head louse egg surface observed by scanning electron microscopy (10,000×). a An egg without nit sheath dissected from anterior oviduct. b An egg with regular nit sheath from a control head louse. c, d Typical irregular shapes of surface of eggs from LNSP1-knockdown head louse. [file 13071_2021_4914_MOESM7_ESM.tif]

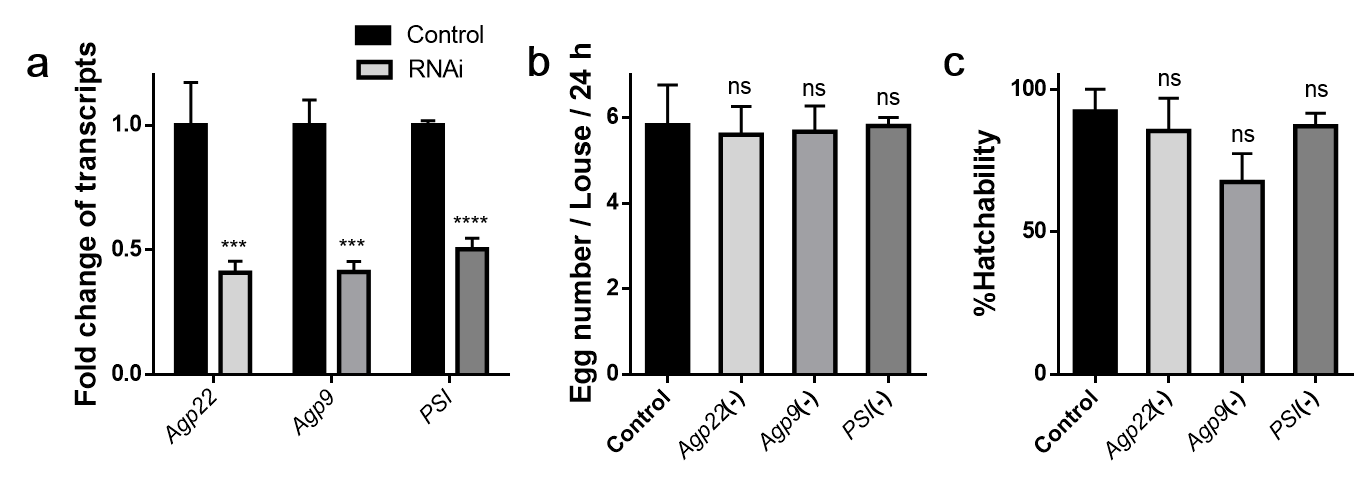

Supplement: Supplementary file 8 — Additional file 8: Figure S6. Effects of RNAi-based knockdown of Agp22, Agp9 or PSI on oviposition. a Relative transcription levels of Agp22, Agp9 and PSI at 72 h post-dsRNA injection. b The number of eggs and c %hatchability of eggs laid from control or knockdown females during 60 ~ 84 h after dsRNA injection. Significant differences were tested using Student’s t-test (a; ***P < 0.001; ****P < 0.0001) or one-way ANOVA (b, c; ns, non-significant). Error bars indicate standard deviation. [file 13071_2021_4914_MOESM8_ESM.tif]
