# Supplementary material for: Characterization of nit sheath protein functions and transglutaminase-mediated cross-linking in the human head louse, Pediculus humanus capitis
Source: Parasit Vectors. 2021 Aug 24;14:425. doi: 10.1186/s13071-021-04914-z (PMC8383413; doi:10.1186/s13071-021-04914-z)
Supplement: Supplementary file 9 — Additional file 9: Table S3. Mortality of iodoacetamide (IAA)-injected female head lice. [file 13071_2021_4914_MOESM9_ESM.docx]

**Table S3.** Mortality of iodoacetamide (IAA)-injected female head lice.

| IAA concentration (mM) | %mortality (average ± SD) |
| --- | --- |
| 0 | 0.0 ± 0 |
| 10 | 10.0 ± 14 |
| 20 | 13.3 ± 12 |
| 40 | 80.0 ± 28 |
| 100 | 93.3 ± 12 |
