# Supplementary material for: Characterization of nit sheath protein functions and transglutaminase-mediated cross-linking in the human head louse, Pediculus humanus capitis
Source: Parasit Vectors. 2021 Aug 24;14:425. doi: 10.1186/s13071-021-04914-z (PMC8383413; doi:10.1186/s13071-021-04914-z)
Supplement: Supplementary file 10 — Additional file 10: Table S4. The total numbers of eggs examined and the proportions of affected eggs by various treatments. [file 13071_2021_4914_MOESM10_ESM.docx]

**Table S4.** The total numbers of eggs examined and the proportions of affected eggs by various treatments.

|  | Effect on egg production | | Effect on hatchability | |
| --- | --- | --- | --- | --- |
|  | Total number of eggs from each experiment  (total number of female) | Eggs/female  (mean/standard deviation) | Viable egg number/total egg number | Average  %Hatchability |
| ***LNSP* knockdown** |  |  |  |  |
| Control | 64 (16), 68 (16), 78 (16), 106 (16), 79 (16) | 4.90 ± 1.03 | 58/60, 63/64, 70/73 | 97.0 ± 1.31 |
| *LNSP1* (-) | 56 (14), 69 (15), 76 (14) | 4.68 ± 0.72 | 4/60, 6/69, 2/67 | 6.12 ± 2.89 |
| *LNSP2* (-) | 4 (16), 28 (16), 14 (16) | 0.96 ± 0.75 | 0/8, 0/13, 0/13 | 0 ± 0 |
| *LNSP1, 2* (-) | 70 (18), 72 (15), 48 (15) | 3.96 ± 0.80 | 0/59, 0/60, 0/48 | 0 ± 0 |
| ***TG* knockdown** |  |  |  |  |
| Control | 56 (8), 45 (10), 51 (18) | 4.78 ± 2.10 | 116/116, 85/85, 90/102, 136/148, 96/106 | 94.1 ± 5.51 |
| *TG* (-) | 58 (10), 63 (20), 50 (22), 26 (12) | 3.35 ± 1.69 | 27/81, 24/66, 30/100 | 33.2 ± 3.18 |
| **GGsTOP injection** |  |  |  |  |
| Control | 86 (16), 102 (18), 80 (14) | 5.59 ± 0.18 | 63/87, 57/63, 45/60, 57/63, 90/90, 84/98, 62/66, 66/84 | 85.8 ± 9.71 |
| GGsTOP | 39 (11), 70 (18), 64 (14) | 4.00 ± 0.52 | 33/72, 27/42, 36/57, 28/40, 45/60, 21/45, 30/54, 24/42 | 59.7 ± 10.4 |
